# Incorporating the synthetic CT image for improving the performance of deformable image registration between planning CT and cone-beam CT

**DOI:** 10.3389/fonc.2023.1127866

**Published:** 2023-02-22

**Authors:** Na Li, Xuanru Zhou, Shupeng Chen, Jingjing Dai, Tangsheng Wang, Chulong Zhang, Wenfeng He, Yaoqin Xie, Xiaokun Liang

**Affiliations:** ^1^ School of Biomedical Engineering, Guangdong Medical University, Dongguan, Guangdong, China; ^2^ Dongguan Key Laboratory of Medical Electronics and Medical Imaging Equipment, Dongguan, Guangdong, China; ^3^ Songshan Lake Innovation Center of Medicine & Engineering, Guangdong Medical University, Dongguan, Guangdong, China; ^4^ Shenzhen Institute of Advanced Technology, Chinese Academy of Sciences, Shenzhen, Guangdong, China; ^5^ Department of Biomedical Engineering, Southern Medical University, Guangzhou, China; ^6^ Department of Radiation Oncology, Beaumont Health, Royal Oak, MI, United States

**Keywords:** synthetic image, deformable image registration (DIR), breast cancer, deep learning, radiation therapy

## Abstract

**Objective:**

To develop a contrast learning-based generative (CLG) model for the generation of high-quality synthetic computed tomography (sCT) from low-quality cone-beam CT (CBCT). The CLG model improves the performance of deformable image registration (DIR).

**Methods:**

This study included 100 post-breast-conserving patients with the pCT images, CBCT images, and the target contours, which the physicians delineated. The CT images were generated from CBCT images *via* the proposed CLG model. We used the Sct images as the fixed images instead of the CBCT images to achieve the multi-modality image registration accurately. The deformation vector field is applied to propagate the target contour from the pCT to CBCT to realize the automatic target segmentation on CBCT images. We calculate the Dice similarity coefficient (DSC), 95 *%* Hausdorff distance (HD95), and average surface distance (ASD) between the prediction and reference segmentation to evaluate the proposed method.

**Results:**

The DSC, HD95, and ASD of the target contours with the proposed method were 0.87 ± 0.04, 4.55 ± 2.18, and 1.41 ± 0.56, respectively. Compared with the traditional method without the synthetic CT assisted (0.86 ± 0.05, 5.17 ± 2.60, and 1.55 ± 0.72), the proposed method was outperformed, especially in the soft tissue target, such as the tumor bed region.

**Conclusion:**

The CLG model proposed in this study can create the high-quality sCT from low-quality CBCT and improve the performance of DIR between the CBCT and the pCT. The target segmentation accuracy is better than using the traditional DIR.

## 1 Introduction

In image-guided radiotherapy, Cone-beam computed tomography (CT) (CBCT) has been incorporated into the contemporary linear accelerators (1–3). However, CBCT has low image quality due to the small number of x-ray projections and the long acquisition time, which impedes the following deformable image registration (DIR) procedure (4). In adaptive radiation treatment (ART), DIR between the planning computed tomography (pCT) and daily CBCT is required (5, 6). The deformable vector field (DVF) generated from the DIR might help with patient setup, contour propagation, target definition, and online dosage computation (7). The sum of squared differences and the mean absolute difference is employed in many traditional DIR methods to assess the fixed and moving image registration performance (8). On the other hand, these measurements presume that the moving and fixed image intensities are consistent. As a result of the image intensity discrepancy, the sum of squared difference (SSD) and Mean absolute error (MAE) cannot be directly used for pCT-CBCT DIR (9–16).

Many studies focused on the deep learning (DL) based DIR (17–21) Kearney et al. developed an unsupervised learning technique to register the CT to CBCT (22). For multimodal CT-CBCT image registration, Fu et al. presented an unsupervised DL registration network that used directional local structural similarity and original images as input. (23). A DL registration model was proposed by Han et al. to predict Organ at Risk (OAR) segmentations on the CBCT based on planned CT segmentation (24). However, the severe artifacts of CBCT greatly limit the deformable image registration accuracy of CBCT with planning CT. Therefore, many scholars have proposed to convert CBCT into high-quality synthetic CT before registration. Fu et al. propose synthesizing a high-quality CT from CBCT to reduce image artifacts and perform intensity correction before image registration (3). The Cycle-GAN was used in the high-quality CT generation. Although Cycle-GAN can improve the quality of CBCT images, it does not mean that it helps to improve the accuracy of registration. one of the more fatal drawbacks of Cycle-GAN is that the anatomical geometry may change after the image quality improvement. These changes include the movement, deformation, or disappearance of anatomical structures, which bring huge errors to the subsequent deformable image registration. Therefore, an urgent need is to investigate a synthetic CT generation model with anatomical geometric consistency.

This study proposes a contrast learning-based generativity (CLG) model for synthetic CT (sCT) generation to address the above issues. The proposed method maintains the consistency of the anatomy after image synthesis. As a result, the synthesis images are more trustworthy than the cycleGAN. In addition, the high-quality synthetic CT improves the deformable image registration performance of CBCT and breast pCT.

## 2 Materials and methods

### 2.1 Data acquisition and processing

The study retrospectively included 100 patients who underwent radiotherapy after breast-conserving surgery. The patients were treated using a standard treatment planning process with CT images and at least one set of CBCT images acquired during treatment. The CT images were acquired using the Siemens Medical System scanner with a voxel size of 0.977 × 0.977 × 5 mm^3^ and a data size of 512 ×512 × 80. The CBCT was acquired using the Varian Edge (Varian Medical Systems, Palo Alto, CA) scanner with a voxel size of 0.977 × 0.977 × 5 mm^3^. Due to the difference in scanning range and voxel size between CT and CBCT, we first rigidly aligned the CT and resampled the voxel size to match CBCT.

In our study, a DL network generated sCT images from CBCT images. And then, pCT was aligned with CBCT and sCT images, respectively, using the DIR method. Next, the contours on pCT were propagated on CBCT (sCT) images. Physicians first manually outlined pCT and CBCT data contours (target area contours including tumor bed area clinical target volume (CTV) 1, CTV 2, Heart). The final contours propagated on the sCT images have a more similar anatomy to the original CBCT, especially in soft tissues with significant effects. The model was trained, validated, and tested using 52/7/41 patients, corresponding to 4160/560/3280 slices.

### 2.2 Synthetic CT generation

The image transformation problem is an untangling problem: the separated content must be preserved between the different image modalities. The appearance must be modified (25, 26). In most cases, the adversarial loss produces the goal appearance, whereas circular consistency loss is used to retain the content (27–29). However, cyclic consistency loss assumes a bijection between two domains, which is usually too restrictive.

**Figure 1:**
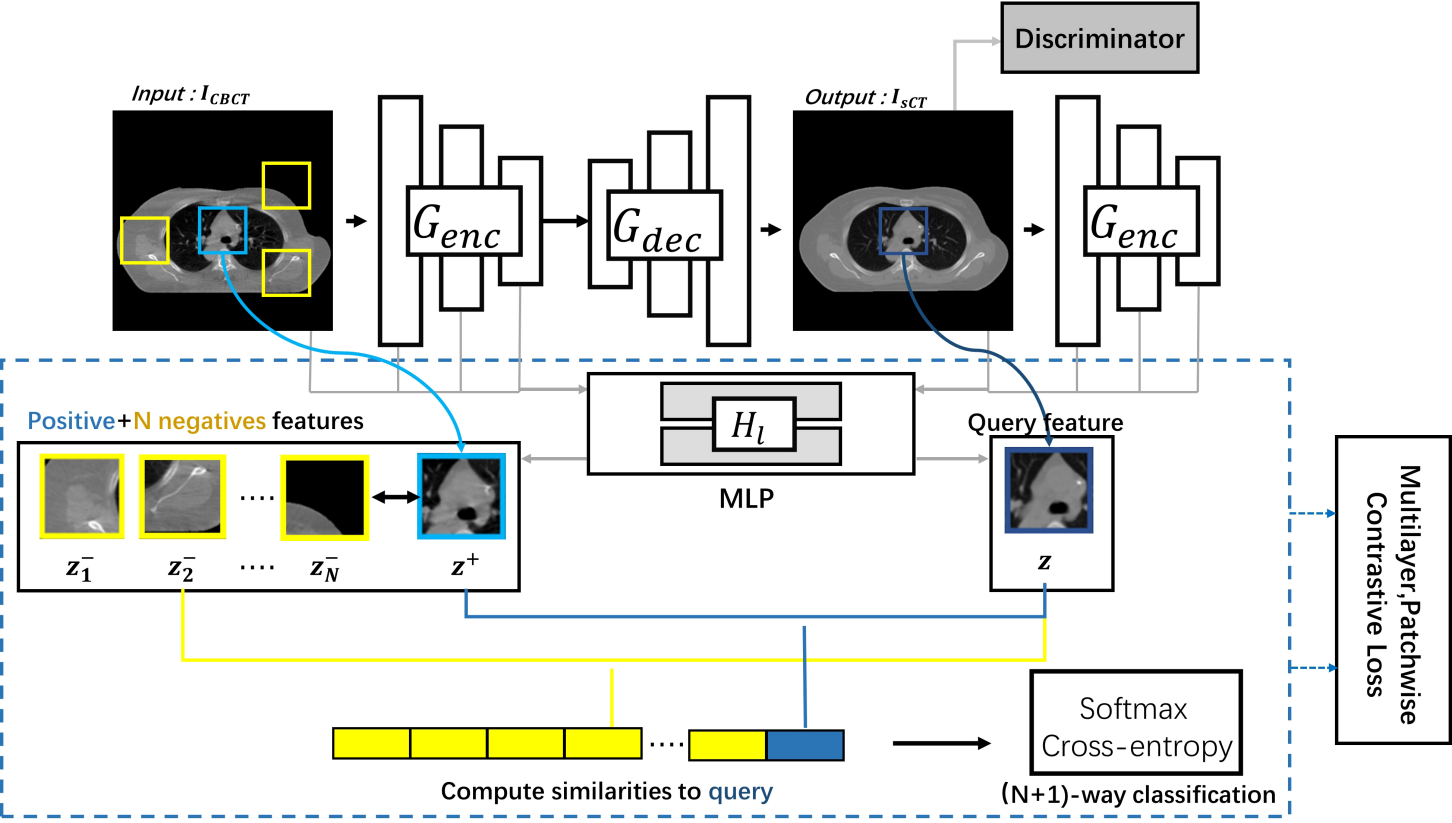
The CLG model network architecture.

In this study, to maintain consistency in content effectively, we used the CLG model to generate the sCT images (30). The CLG model network architecture is schematically shown in **Figure 1**.

The CLG model uses only one-way learning mapping. The I_CBCT_ was the input image, and the I_sCT_ was the output image. Split the generator G into the decoder G_dec_ and the encoder G_enc_ to get the output image. We employed a Resnet-based generator in particular (31). We refer to the encoder as the generator’s first half, and the decoder corresponds to the back half of the generator. The whole image should have an identical structure. Therefore, we should use the learning target of multi-layer image blocks. The feature layers were encoded by an encoder G_enc_, where different layers with different spatial locations represent different image blocks. To generate a sequence of features, we select the L layer feature map $\{{\text{z}}_{\text{l}}right{\}}_{\text{L}}={\left\{{\text{H}}_{\text{l}}\left({\text{G}}_{\text{enc}}^{\text{l}}\left(\text{x}\right)\right)\right\}}_{\text{L}}$ and feed it through the two-layer Multi-layer perceptron (MLP) network H_1_. The number of channels per layer was C_1_. The output y was encoded into the ${\left\{{\hat{\text{z}}}_{\text{l}}\right\}}_{\text{L}}={\left\{{\text{H}}_{\text{l}}({\text{G}}_{\text{enc }}^{\text{l}}(\hat{\text{y}}))\right\}}_{\text{L}}$ in the same way.

After getting the features *via* the MLP network, we introduce contrast learning. The features of the output image become the query samples, the input’s corresponding location features become the positive samples, and the other input’s features become the negative samples. The purpose of contrast learning was to make the query sample and the positive sample signal correlate with the negative sample to form a contrast.

The queries, positive samples, and N negative samples are fixed to map to the Kth dimensional vector *v*, *v* ^+^∈ *ℝ* ^K^ and *v* ^−^∈ *ℝ* ^N×K^, respectively. The n-th negative value is denoted by $v_{n}^{-}\in {\mathbb{R}}^{\text{K}}$ . Our objective aims to link the output and the input image. The query indicates the output image. The corresponding and non-corresponding inputs are positive and negative, respectively. We normalize the vector to the unit sphere to prevent spatial collapse or expansion. The classification problem was built up in an (N+1)-way configuration, with the distance between the query and the other instances scaled by the temperature of 0.07 (32, 33). The chance of choosing a positive example among negative instances was determined using the cross-entropy loss.


(1) $$\ell \left(v,v^{+},v^{-}\right)=-\log\;\left[\frac{exp\;\left(v\cdot \frac{v^{+}}{\tau }\right)}{exp\;\left(v\cdot \frac{v^{+}}{\tau }\right)+\sum_{n=1}^{N}exp\;\left(v\cdot \frac{v_{n}^{-}}{\tau }\right)}\right]$$

Finally, we obtain the loss of multi-layer patch contrast learning:


(2) $${ℒ}_{\text{PatchNCE}}(G,H,X)=E_{\text{x}∼\text{X}}\sum_{\text{l}=1}^{\text{L}}\sum_{\text{s}=1}^{{\text{S}}_{\text{l}}}\ell {\left({\hat{\text{z}}}_{1}^{\text{s}},{\text{z}}_{1}^{\text{s}},{\text{z}}_{1}^{\text{S}/\text{s}}\right)}^{L}$$

(3) $${ℒ}_{\text{GAN}}\left(\text{G},\text{D},\text{X},\text{Y}\right)=E_{\text{y}∼\text{Y}}\log\text{ D}\left(\text{y}\right)+E_{\text{x}∼\text{X}}\log\;(1-\text{D}\left(\text{G}\left(\text{x}\right)\right))\;\;\;\;\;\;\;\;\;\;\;\;\;\;\;\;\;\;\;\;\;\;\;\;\;$$

Similarly, an identity contrast loss can be obtained similarly to fix the output image. Where z s 1 and ^z S\s^ l from the input’s first layer feature map from the output’s first layer feature map. The adversarial loss encourages the output to resemble the image in the target domain in terms of appearance (26).

In summary, the total loss function of the Cut network is shown in equation (4) below.


(4) $$\text{ Loss }={ℒ}_{\text{GAN}}\left(\text{G},\text{D},\text{X},\text{Y}\right)+\lambda_{\text{X}}{ℒ}_{\text{PatchNCE }}\left(\text{G},\text{H},\text{X}\right)+\lambda_{\text{Y}}{ℒ}_{\text{PatchNCE }}\left(\text{G},\text{H},\text{Y}\right)$$

where during training, we set *λ*_X_ = *λ*_Y_ = 1.

The learning rate was set to 2*10^-4^, and the Adam optimizer was used. The number of iterations was set to 200 epochs, and the learning rate of the first 50 epochs remained unchanged, while the learning rate of the rest 150 epochs decayed to 0. The model was trained and tested on an NVIDIA 1080 GPU with 8 GB of memory with a batch size of one. The model was based on the PyTorch framework.

### 2.3 Deformable image registration

We used the DiffeoDemons (34) algorithm for the deformation registration algorithm. The Demons-based differential homogeneous registration algorithm solves transformations in the logarithmic domain. The basic concept behind the approach is to represent the current transformation as an index of the smooth velocity field V. We use the homogeneous differential demon to quickly compute *φ* ∘ *exp* (*v*)=*exp* (*V*) ∘ *exp* (*v*) and then update v. The exponential mapping in the Lie algebra (vectorspace of the velocity field) is denoted by the symbol exp. The following equation yields the functional energy:


(5) $$\begin{array}{cc} \text{E}\left(\varphi \right) & =\text{SSD }\left({\text{I}}_{\text{m}},{\text{I}}_{\text{f}},\varphi \right)+\lambda \|\nabla \varphi {\|}^{2}\\ & ={\text{I}}_{\text{m}}\circ \varphi -{\text{I}}_{f}^{2}+\lambda \|\nabla \varphi {\|}^{2} \end{array}$$

where *λ* > 0. It’s worth noticing that the transformation of y’s Jacobian matrix is ∇*φ* . The DiffeoDemons model guarantees a smooth displacement field at all times. I_m_ stands for moving image and I*_hrmf_* stands for the fixed image.

**Figure 2:**
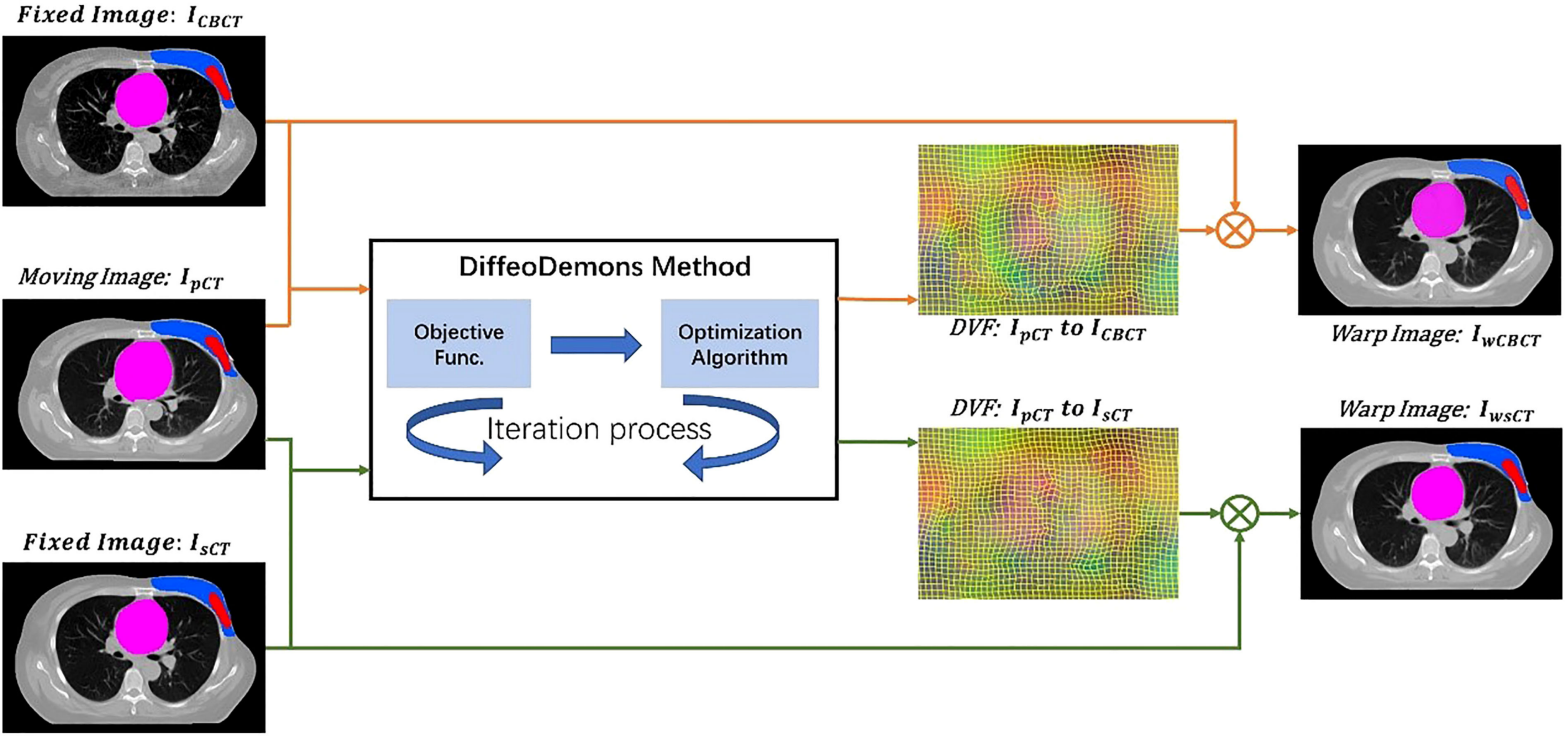
The image registration process with two different methods.

**Figure 2** shows the image registration process with two different methods. To compare the performance of the method with and without the incorporation of the sCT, we obtained the deformable vector field (DVF) of I_pCT_ - *mI*_CBCT_ and I_pCT_ - I_sCT_ by DiffeoDemons deformation registration. The DVFs are used to warp the moving image and the corresponding target labels to obtain the warped CBCT images I*_mwCBCT_*, the warped sCT images I_wsCT_ and the warped target labels.

## 3 Results and discussion

**Figure 3:**
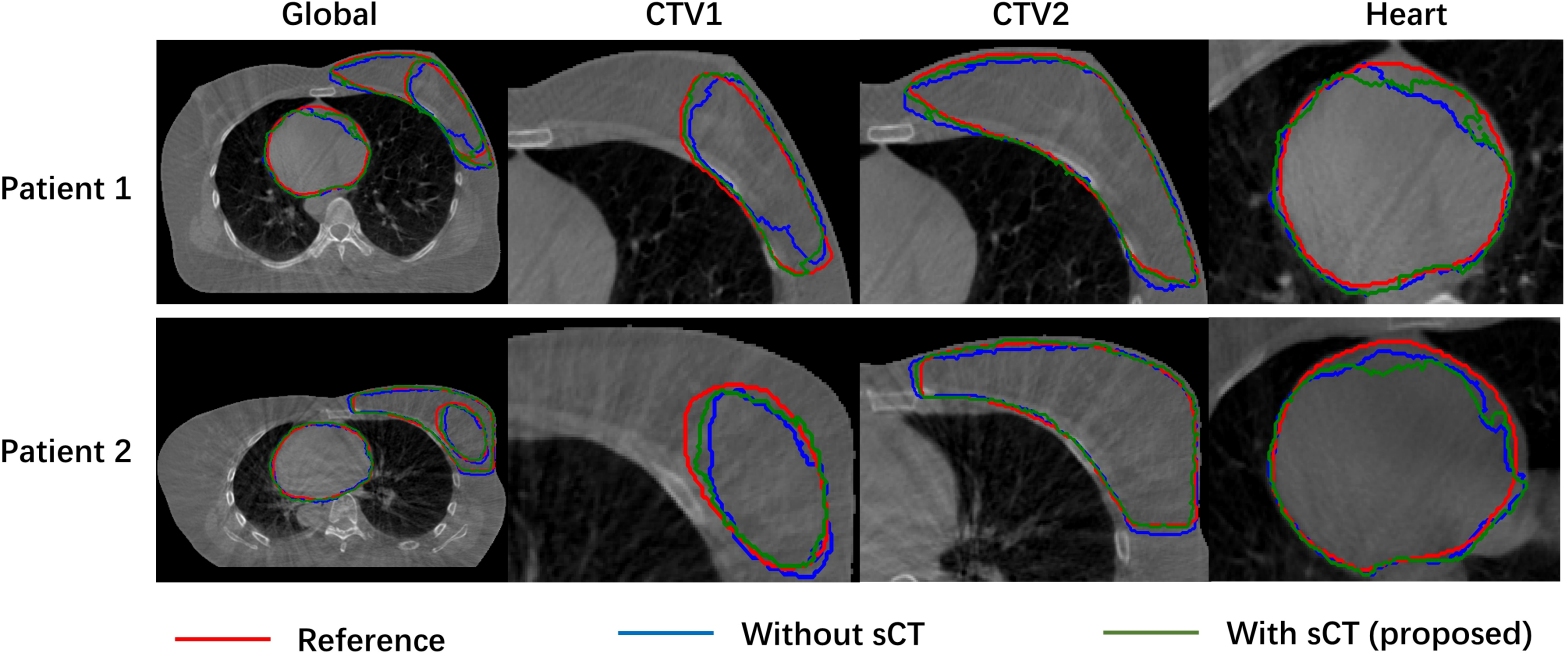
Comparison of object segmentation performance of fixed image using CBCT image and sCT image.

**Figure 4:**
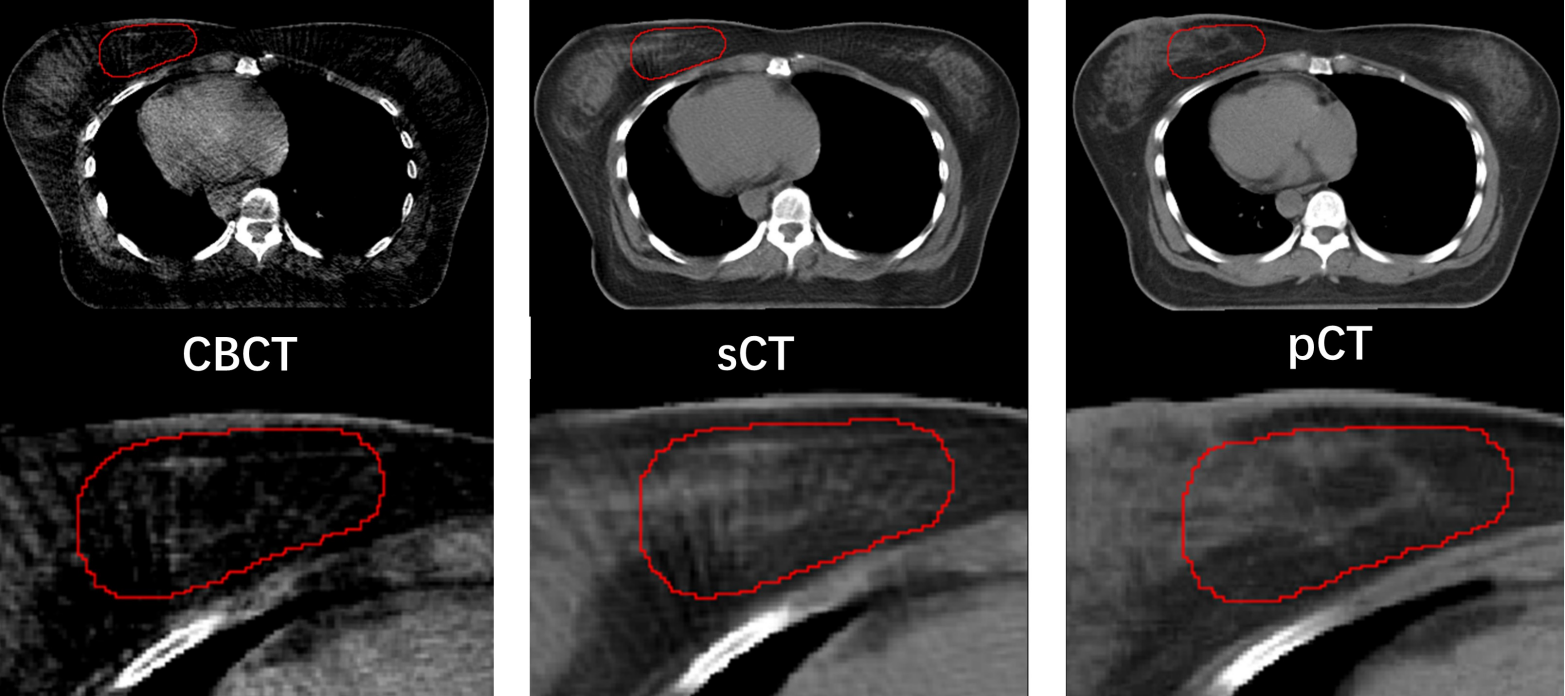
Comparison of soft tissue from CBCT, sCT, and pCT images with same window width and location, with tumor bed areas represented by the red contour line.

**Figure 3** compared the target segmentation performance between the fixed image using the CBCT image and sCT image. The red, blue, and green contours represent the target area on the I_wCBCT_ image and I_wsCT_ image. After using sCT images for fixed image alignment, the target contours are better than the original CBCT images for fixed image alignment. The proposed method can alleviate the motion and scatter artifacts in the patient’s breast CBCT images, improving the performance of the DIR. In the original CBCT, the image contrast of the soft tissues (tumor bed, CTV) was poor and impeded the accurate DIR. **Figure 4** shows the soft tissue comparison of CBCT, sCT, and pCT images at the same window width and position, with the red contours indicating the tumor bed area. The sCT improves the image quality and spatial uniformity while keeping the imaging anatomy unchanged, resembling the tissue information distribution of pCT images. The proposed CLG model can greatly improve the tissue contrast of CBCT images. Thus, the soft tissue segmentation performance can be achieved more accurately.

**Figure 5:**
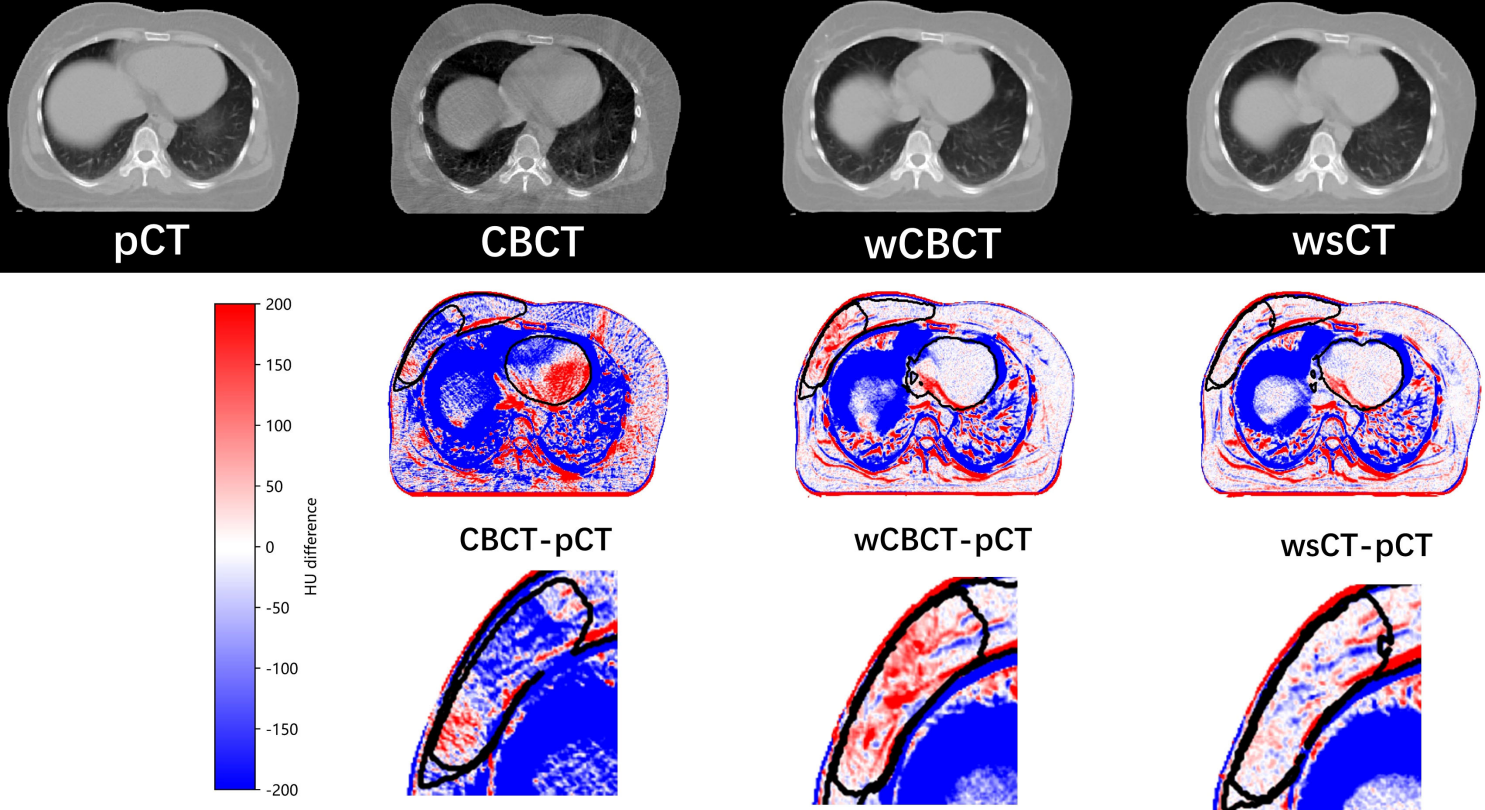
The pCT, CBCT, warped CBCT, and warped sCT images displayed in the same window for a single patient, and the second row shows the difference between CBCT, warped CBCT, and warped sCT images and pCT images.

The first row in **Figure 5** shows the pCT, CBCT, warped CBCT, and warped sCT images displayed in the same window for a single patient, and the second row shows the difference between CBCT, warped CBCT and warped sCT images and pCT images. Since thepatient had significant weight loss, tumor shrinkage, and the influence of respiratory factors during the fractionated treatment, the difference between the pCT and the CBCT is obvious, especially in the lungs. In contrast, the difference between wCBCT-pCT and wsCT-pCT images is significantly reduced, which indicates that the artifact in the CBCT was reduced. The black contour lines represent CTV1, CTV2, and heart target areas in the different maps. The comparison reveals that the difference between westand pCT images in the target area region is smaller than between wCBCT and pCT images. Furthermore, in the soft tissue region, such as the tumor bed, the image intensity difference between the registration synthetic computed tomography and pCT is smaller, which indicates that the sCT generated by the CLG model proposed in this paper can improve the image contrast of the soft tissue. Therefore, the sCT’s image quality is comparable to that of the pCT and benefits the performance of the DIR.

**Figure 6:**
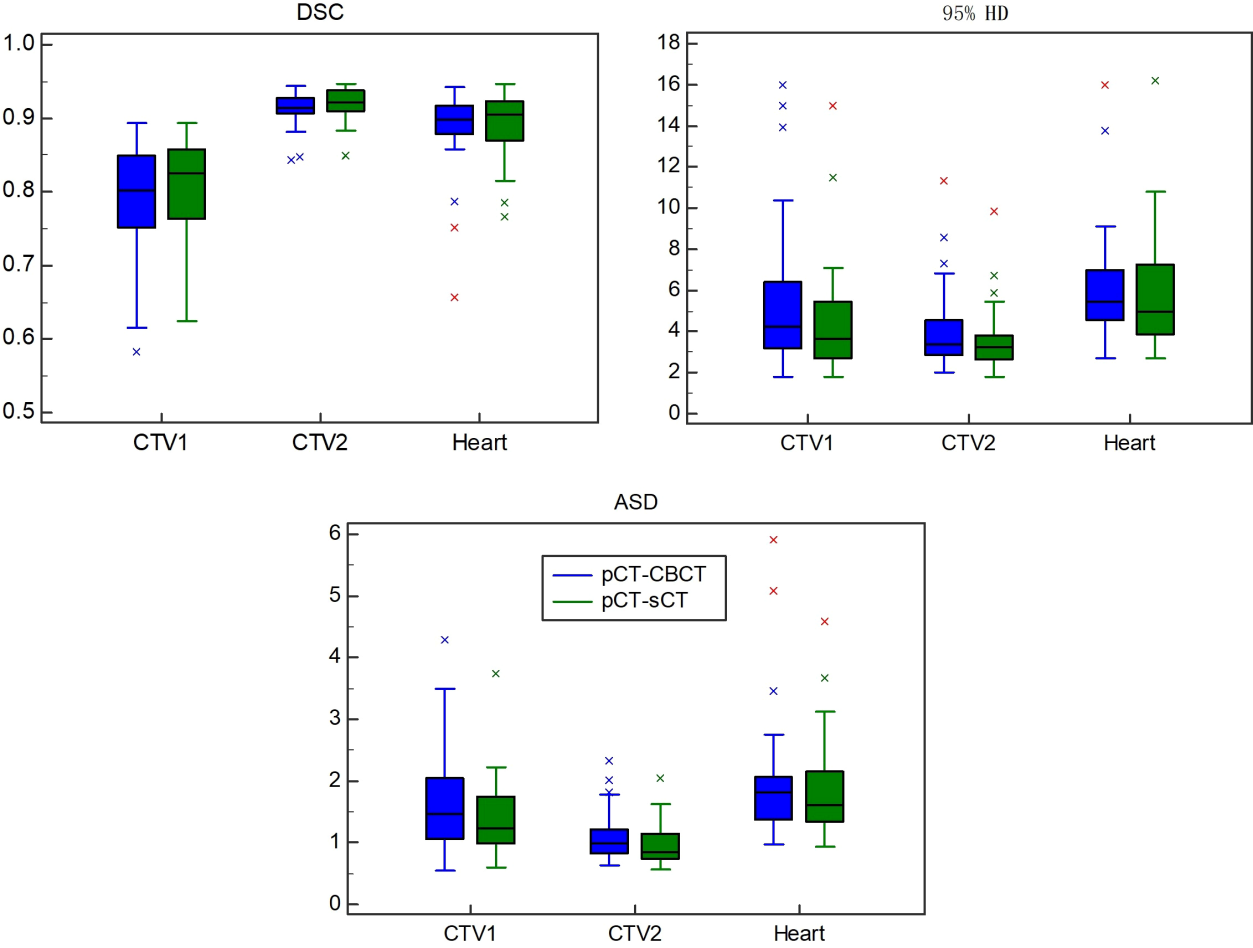
The results of the Dice similarity coefficient (DSC), 95 percent Hausdorff distance (HD95), and average surface distance (ASD) on the various techniques.

The results of the Dice similarity coefficient (DSC), 95 percent Hausdorff distance (HD95), and average surface distance (ASD) on the various techniques are shown in **Table 1** and **Figure 6**. Compared with the traditional method using the CBCT image as the fixed image, the proposed method clearly shows that incorporating the sCT image in the DIR achieved a better result in DSC, HD95, and ASD. For example, the DSC value of pCT to sCT in the CTV1 (tumor bed) is 0.81 ± 0.06, while the DSC value of pCT to CBCT is only 0.79 ± 0.08.

**Table 1:** The results of the Dice similarity coefficient (DSC), 95 percent Hausdorff distance (HD95), and average surface distance (ASD) on the various techniques.

				pCT-sCT		
pCT-CBCT	DSC	HD95(mm)	ASD(mm)	DSC	HD95(mm)	ASD(mm)
CTV1	0.79 ± 0.08	5.45 ± 3.38	1.61 ± 0.82	0.81 ± 0.06	4.39 ± 2.43	1.38 ± 0.60
CTV2	0.91 ± 0.02	3.98 ± 1.82	1.09 ± 0.39	0.92 ± 0.02	3.47 ± 1.47	0.98 ± 0.33
Heart	0.89 ± 0.05	6.07 ± 2.59	1.94 ± 0.95	0.89 ± 0.04	5.80 ± 2.63	1.88 ± 0.76
Average	0.86 ± 0.05	5.17 ± 2.60	1.55 ± 0.72	0.87 ± 0.04	4.55 ± 2.18	1.41 ± 0.56

Breast cancer radiation therapy is based on the pCT for treatment planning. However, the target area and anatomy will change with the treatment process. In addition, positional errors and patient respiration can result in underdose to the target area and increased dose to normal organs. ART uses daily CBCT images to analyze changes in the target area and anatomy. Correction of anatomical changes is achieved by the DIR method. The DIR between the CBCT and pCT image is a multimodal DIR problem. The multimodal DIR is challenging because establishing effective similarity measures between regions or features of multimodal images is difficult due to the nonlinear variation of grayscale features. To this end, this study achieves accurate DIR of pCT and fractional CBCT of breast cancer and accurate propagation of the corresponding target region contours by converting the multimodal DIR problem into a unimodal problem.

Traditional image synthesis methods usually use cycleGAN. cycleGAN is good at suppressing artifacts, but it does not mean that the generated images are reliable. The anatomy changes before and after the improvement of CBCT image quality. These changes include shifting, distortion, or disappearance of the target area, which brings huge errors to the subsequent automatic target area localization. These changes include the movement, distortion, or disappearance of the target area, which can lead to significant errors in subsequent DIR. We proposed a multi-level contrast learning-based approach for generating quantitative CBCT images with anatomical geometric consistency to improve the quality of CBCT images. Based on the generative adversarial network, we introduce a contrast-loss function to ensure the consistency of the anatomical structure. The proposed loss function discards the cyclic consistency loss function in cycleGAN and avoids the strong mapping relationship brought by the cyclic consistency loss function. As a result, it not only improves the computational speed but also better in image generation details. The advantage of our method is that it maintains consistent anatomical geometry before and after image generation. As a result, the generated images are more trustworthy.

This study used the CLG model to generate high-quality sCT from CBCT. A patch loss was proposed based on contrast learning to calculate the similarity between patches, which enables learning the CT value distribution of pCT without changing the anatomical structure of CBCT images. The proposed CLG model can alleviate the effects of low contrast, high noise, and artifact contamination in soft tissues. We performed multimodal pCT-CBCT DIR and unimodal pCT-sCT DIR by the DiffeoDemons algorithm. The target segmentation results show that the unimodal pCT-sCT registration is significantly better than multimodal pCT-CBCT registration. In this paper, only the DiffeoDemons algorithm was used to perform DIR; however, many other DIR algorithms can be explored for potential performance improvements (35).

Although the proposed framework produces accurate results by incorporating the sCT, further refinements may be made. Firstly, we adopted the patch-based unsupervised convolutional, which is computationally intensive. The training step may increase efficiency by balancing network breadth, depth, and resolution. Second, more training images may be needed to ensure the findings’ correctness. We will expand the datasets with a wide range of anatomic variants and from other CT scanners in the future to improve the network’s resilience. This study used 8000 CT slices from 100 patients to evaluate the model. The outcomes are deemed clinically satisfactory. The prediction accuracy will increase, and the network will avoid potential overfitting when the training dataset is expanded.

## 4 Conclusion

This work proposed a CLG model to create high-quality sCT from CBCT. Instead of the CBCT images with severe artifacts, the pCT performs DIR with high-quality sCT images for the target contour propagation. The results showed that incorporating the sCT Image can improve the performance of DIR between pCT and CBCT, especially in soft tissues. Furthermore, the proposed method is quite general and can be applied to other organs, such as the abdomen and prostate.

## Data availability statement

The hospital datasets are protected for patient privacy and are not publicly available. However, the datasets are available from the corresponding author upon reasonable request.

## Author contributions

NL, XZ, YX, and XL contributed to the conception and design of the study. NL, XZ, SC organized the database. XZ, JD performed the statistical analysis. XZ XL wrote the first draft of the manuscript. NL, XZ, TW, CZ, WH, and XL wrote sections of the manuscript. All authors contributed to the manuscript revision and read and approved the submitted version.

## References

[1] Létourneau D Wong JW Oldham M Gulam M Watt L Jaffray DA. Cone-beam-ct guided radiation therapy: technical implementation. Radiother Oncol (2005) 75:279–86. doi: 10.1016/j.radonc.2005.03.001 15890424

[2] Ding GX Duggan DM Coffey CW. Characteristics of kilovoltage x-ray beams used for cone-beam computed tomography in radiation therapy. Phys Med Biol (2007) 52:1595. doi: 10.1088/0031-9155/52/6/004 17327651

[3] Fu Y Lei Y Liu Y Wang T Curran WJ Liu T. Cone-beam computed tomography (cbct) and ct image registration aided by cbct-based synthetic ct. In: Medical imaging 2020: Image processing, vol. 11313. Houston, Texas, United States: SPIE (2020). p. 721–7.

[4] Xu Y Yan H Ouyang L Wang J Zhou L Cervino L. A method for volumetric imaging in radiotherapy using single x-ray projection. Med Phys (2015) 42:2498–509. doi: 10.1118/1.4918577 PMC4409629 25979043

[5] Lawson JD Schreibmann E Jani AB Fox T. Quantitative evaluation of a cone-beam computed tomography–planning computed tomography deformable image registration method for adaptive radiation therapy. J Appl Clin Med Phys (2007) 8:96–113. doi: 10.1120/jacmp.v8i4.2432 18449149 PMC5722621

[6] Liang X Chen L Nguyen D Zhou Z Gu X Yang M. Generating synthesized computed tomography (ct) from cone-beam computed tomography (cbct) using cyclegan for adaptive radiation therapy. Phys Med Biol (2019) 64:125002. doi: 10.1088/1361-6560/ab22f9 31108465

[7] Loi G Fusella M Lanzi E Cagni E Garibaldi C Iacoviello G. Performance of commercially available deformable image registration platforms for contour propagation using patient-based computational phantoms: a multi-institutional study. Med Phys (2018) 45:748–57. doi: 10.1002/mp.12737 29266262

[8] Fu Y Lei Y Wang T Curran WJ Liu T Yang X. Deep learning in medical image registration: a review. Phys Med Biol (2020) 65:20. doi: 10.1088/1361-6560/ab843e PMC7759388 32217829

[9] Brock KK Dawson LA Sharpe MB Moseley DJ Jaffray DA. Feasibility of a novel deformable image registration technique to facilitate classification, targeting, and monitoring of tumor and normal tissue. Int J Radiat Oncol Biol Phys (2006) 64:1245–54. doi: 10.1016/j.ijrobp.2005.10.027 16442239

[10] Chao KC Bhide S Chen H Asper J Bush S Franklin G. Reduce in variation and improve efficiency of target volume delineation by a computer-assisted system using a deformable image registration approach. Int J Radiat Oncol Biol Phys (2007) 68:1512–21. doi: 10.1016/j.ijrobp.2007.04.037 17674982

[11] Kaus MR Brock KK Pekar V Dawson LA Nichol AM Jaffray DA. Assessment of a model-based deformable image registration approach for radiation therapy planning. Int J Radiat Oncol Biol Phys (2007) 68:572–80. doi: 10.1016/j.ijrobp.2007.01.056 17498570

[12] Lee C Langen KM Lu W Haimerl J Schnarr E Ruchala KJ. Assessment of parotid gland dose changes during head and neck cancer radiotherapy using daily megavoltage computed tomography and deformable image registration. Int J Radiat Oncol Biol Phys (2008) 71:1563–71. doi: 10.1016/j.ijrobp.2008.04.013 18538505

[13] Wang H Garden AS Zhang L Wei X Ahamad A Kuban DA. Performance evaluation of automatic anatomy segmentation algorithm on repeat or four-dimensional computed tomography images using deformable image registration method. Int J Radiat Oncol Biol Phys (2008) 72:210–9. doi: 10.1016/j.ijrobp.2008.05.008 PMC2593788 18722272

[14] Reed VK Woodward WA Zhang L Strom EA Perkins GH Tereffe W. Automatic segmentation of whole breast using atlas approach and deformable image registration. Int J Radiat Oncol Biol Phys (2009) 73:1493–500. doi: 10.1016/j.ijrobp.2008.07.001 PMC2729433 18804333

[15] Rigaud B Simon A Castelli J Gobeli M Ospina Arango J-D Cazoulat G. Evaluation of deformable image registration methods for dose monitoring in head and neck radiotherapy. BioMed Res Int (2015) 2015:726268. doi: 10.1155/2015/726268 25759821 PMC4339705

[16] Polan DF Feng M Lawrence TS Ten Haken RK Brock KK. Implementing radiation dose-volume liver response in biomechanical deformable image registration. Int J Radiat Oncol Biol Phys (2017) 99:1004–12. doi: 10.1016/j.ijrobp.2017.06.2455 PMC5654673 28864401

[17] Chen G Lin Y Elder E Ghavidel B McDonald M Langen K. Airnet: fused analytical and iterative image reconstruction method with deep learning regularization for high-quality sparse-data on-board cbct. Int J Radiat Oncol Biol Phys (2020) 108:e305. doi: 10.1016/j.ijrobp.2020.07.729

[18] Liang X Zhao W Hristov DH Buyyounouski MK Hancock SL Bagshaw H. A deep learning framework for prostate localization in cone beam ct-guided radiotherapy. Med Phys (2020) 47:4233–40. doi: 10.1002/mp.14355 PMC10823910 32583418

[19] Liang X Li N Zhang Z Xiong J Zhou S Xie Y. Incorporating the hybrid deformable model for improving the performance of abdominal ct segmentation *via* multi-scale feature fusion network. Med Image Anal (2021) 73:102156. doi: 10.1016/j.media.2021.102156 34274689

[20] Jiang Y Liang X Han Z Wang W Xi S Li T. Radiographical assessment of tumour stroma and treatment outcomes using deep learning: a retrospective, multicohort study. Lancet Digital Health (2021) 3:e371–82. doi: 10.1016/S2589-7500(21)00065-0 34045003

[21] Jiang Y Liang X Wang W Chen C Yuan Q Zhang X. Noninvasive prediction of occult peritoneal metastasis in gastric cancer using deep learning. JAMA Network Open (2021) 4:e2032269–e2032269. doi: 10.1001/jamanetworkopen.2020.32269 33399858 PMC7786251

[22] Kearney V Haaf S Sudhyadhom A Valdes G Solberg TD. An unsupervised convolutional neural network-based algorithm for deformable image registration. Phys Med Biol (2018) 63:185017. doi: 10.1088/1361-6560/aada66 30109996

[23] Fu Y Lei Y Wang T Zhou J Patel P Curran WJ. Abdominal ct-cbct deformable image registration using deep neural network with directional local structural similarity. In: Medical imaging 2021: Image-guided procedures, robotic interventions, and modeling, vol. 11598. California, United States: SPIE (2021). p. 343–8.

[24] Han X Hong J Reyngold M Crane C Cuaron J Hajj C. Deep-learning-based image registration and automatic segmentation of organs-at-risk in cone-beam ct scans from high-dose radiation treatment of pancreatic cancer. Med Phys (2021) 48:3084–95. doi: 10.1002/mp.14906 PMC9282672 33905539

[25] Isola P Zhu J-Y Zhou T Efros AA. Image-to-image translation with conditional adversarial networks. In: Proceedings of the IEEE conference on computer vision and pattern recognition Honolulu, HI, USA: IEEE (2017). p. 1125–34.

[26] Goodfellow I Pouget-Abadie J Mirza M Xu B Warde-Farley D Ozair S. Generative adversarial networks. Commun ACM (2020) 63:139–44. doi: 10.1145/3422622

[27] Kim T Cha M Kim H Lee JK Kim J. Learning to discover cross-domain relations with generative adversarial networks. In: International conference on machine learning. Sydney, NSW, Australia: PMLR (2017). p. 1857–65.

[28] Yi Z Zhang H Tan P Gong M. Dualgan: Unsupervised dual learning for image-to-image translation. In: Proceedings of the IEEE international conference on computer vision Venice, Italy: IEEE (2017). p. 2849–57.

[29] Zhu J-Y Park T Isola P Efros AA. Unpaired image-to-image translation using cycle-consistent adversarial networks. In: Proceedings of the IEEE international conference on computer vision. Venice, Italy: IEEE (2017). p. 2223–32.

[30] Park T Efros AA Zhang R Zhu J-Y. Contrastive learning for unpaired image-to-image translation. In: European Conference on computer vision. Glasgow, UK: Springer (2020). p. 319–45.

[31] Johnson J Alahi A Fei-Fei L. Perceptual losses for real-time style transfer and super-resolution. In: European Conference on computer vision. Amsterdam, The Netherlands: Springer (2016). p. 694–711.

[32] He K Fan H Wu Y Xie S Girshick R. Momentum contrast for unsupervised visual representation learning. In: Proceedings of the IEEE/CVF conference on computer vision and pattern recognition. (Virtual conference) IEEE (2020). p. 9729–38.

[33] Wu Z Xiong Y Yu SX Lin D. Unsupervised feature learning via non-parametric instance discrimination. In: Proceedings of the IEEE conference on computer vision and pattern recognition. Salt Lake City, USA: IEEE (2018). p. 3733–42.

[34] Yang D Li H Low DA Deasy JO Naqa IE. A fast inverse consistent deformable image registration method based on symmetric optical flow computation. Phys Med Biol (2008) 53:6143–65. doi: 10.1088/0031-9155/53/21/017 PMC3915046 18854610

[35] Heinrich HP Jenkinson M Brady M Schnabel JA. Mrf-based deformable registration and ventilation estimation of lung ct. IEEE Trans Med Imaging (2013) 32:1239–48. doi: 10.1109/TMI.2013.2246577 23475350

